# Evaluation of CdZnTeSe as a high-quality gamma-ray spectroscopic material with better compositional homogeneity and reduced defects

**DOI:** 10.1038/s41598-019-43778-3

**Published:** 2019-05-13

**Authors:** Utpal N. Roy, Giuseppe S. Camarda, Yonggang Cui, Rubi Gul, Ge Yang, Jakub Zazvorka, Vaclav Dedic, Jan Franc, Ralph B. James

**Affiliations:** 1 Brookhaven National Laboratory, Upton, NY 11973 USA; 2 Institute of Physics, Charles University, Ke Karlovu 5, Prague, 121 16 Czech Republic; 3 Present Address: North Carolina State University, Raleigh, NC 27695-7909 USA; 4 Present Address: Savannah River National Laboratory, Aiken, SC 29808 USA

**Keywords:** Materials for devices, Electronic devices

## Abstract

X- and gamma-ray detectors have broad applications ranging from medical imaging to security, non-proliferation, high-energy physics and astrophysics. Detectors with high energy resolution, e.g. less than 1.5% resolution at 662 keV at room temperature, are critically important in most uses. The efficacy of adding selenium to the cadmium zinc telluride (CdZnTe) matrix for radiation detector applications has been studied. In this paper, the growth of a new quaternary compound Cd_0.9_Zn_0.1_Te_0.98_Se_0.02_ by the Traveling Heater Method (THM) is reported. The crystals possess a very high compositional homogeneity with less extended defects, such as secondary phases and sub-grain boundary networks. Virtual Frisch-grid detectors fabricated from as-grown ingots revealed ~0.87–1.5% energy resolution for 662-keV gamma rays. The superior material quality with a very low density of defects and very high compositional homogeneity heightens the likelihood that Cd_0.9_Zn_0.1_Te_0.98_Se_0.02_ will be the next generation room-temperature detector material.

## Introduction

Radiation detectors are critically needed in applications of medical imaging, security, non-proliferation, astrophysics and high-energy physics^1–7^. All these applications demand high-performance detectors at a reasonably low cost. Most radiation detector materials are categorized into two classes, scintillators and semiconductors. Semiconductor-based detectors outperform scintillators in terms of efficiency per unit volume, energy resolution and proportionality of gamma-ray energies and pulse height.

The requirements for semiconductor materials in X- and gamma-ray detector applications are very stringent. An ideal material should be composed of elements with high atomic number to ensure sufficient absorption of gamma rays; its band-gap should be high to fulfill the requirement of high resistivity of the material at room temperature to achieve low dark current without cooling; and, the material should have excellent charge-transport characteristics to ensure full charge collection. As opposed to semiconductor sensors for low-energy photons, radiation detectors need to be thick enough to ensure sufficient absorption of high-energy X and gamma rays within the active volume of the detector. Thus, detector materials should have the lowest possible defects (charge trapping centers), so that the induced charge carriers can travel through the entire length of the detector without being trapped. In addition, to produce detectors at low cost, materials ought to be produced with high production yield, requiring high compositional uniformity in the as-grown materials. To meet these challenges, researchers have been exploring suitable radiation detector materials for more than four decades. So far, only a handful of materials evolved as prospective candidates^8–13^ such as HgI_2_, CdTe, CdZnTe and TlBr. Very recently, CsPbBr_3_ (3.8% at 662 keV) was reported to be a promising material^14^ for this purpose, but its energy resolution still needs to be improved. Among these materials, cadmium zinc telluride (Cd_0.9_Zn_0.1_Te, CZT) is still the most successful material that is being used commercially. In the last three decades, intense efforts have been invested to improve the material properties of CZT. However, CZT technology still suffers from three major detrimental defects including compositional inhomogeneity due to the non-unity segregation coefficient (~1.35)^15^ of Zn, the presence of a high concentration of secondary phases, and high concentrations of sub-grain boundaries/dislocation walls in the CZT matrix. Thus, the production yield of CZT suffers from the compositional inhomogeneity, while the secondary phases and the sub-grain boundary networks severely affect the charge collection and hence degrade the spectral response^16,17^. These defects result in the high cost of CZT materials and limit the widespread deployment of the technology.

As compared to other melt-growth methods, mainly the Bridgman growth technique, the traveling heater method (THM) is the most common means to grow CZT crystals commercially. The main detriment of THM-grown CZT ingots is that wafers need post-growth annealing to achieve detector-grade quality^18^. This additional process further increases the production cost of the material. Recently we observed that the addition of selenium in the CdTe matrix greatly reduces the sub-grain boundary network and secondary-phase concentration, and thus improves the compositional uniformity^19–21^.

To solve the issues presently limiting the deployment of CZT technology, the advantages of adding Se to the CZT matrix were explored. Here we report the growth of Cd_0.9_Zn_0.1_Te_0.98_Se_0.02_ (CZTS) by the traveling heater method. The addition of Se was found to have profound effects in reducing the sub-grain boundary network and the secondary-phase concentration. Furthermore, the achieved compositional homogeneity in both axial and radial directions was observed over about 90% of the ingot volume. Virtual Frisch-grid CZTS detectors were fabricated from as-grown CZTS crystals; outstanding energy resolutions of ~0.87% at 662 keV were achieved without reliance on pulse-processing algorithms to correct for electron trapping. Hence, we can successfully eliminate the need for a post-growth annealing process as used extensively for THM-grown CZT, while also achieving excellent transport properties.

## Results

**Figure 1:**
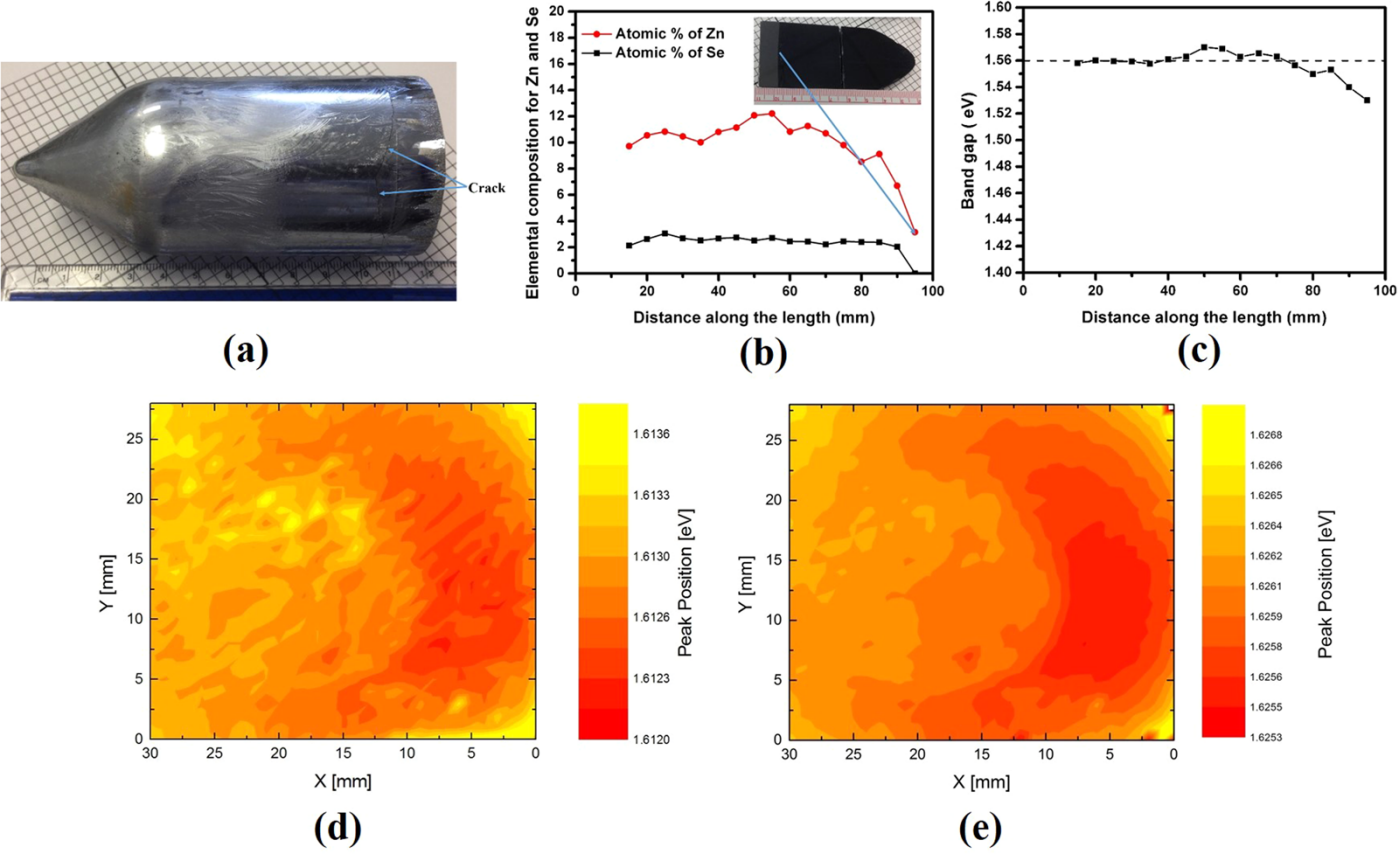
(**a**) As-grown Cd_0.9_Zn_0.1_Te_0.98_Se_0.02_ ingot with 52-mm diameter, grown by the THM, (**b)** Variation of Zn and Se (atomic %) along the length of the ingot, (**c**) Calculated band-gap along the length of the ingot. The inset shows the wafer cut along the length of the as-grown Cd_0.9_Zn_0.1_Te_0.98_Se_0.02_ ingot, and the map of PL peak energy positions at 7 K for the as-grown Cd_0.9_Zn_0.1_Te_0.98_Se_0.02_ wafer cut perpendicular to the growth axis, (**d)** (A°, X) peak mapping and (**e)** (D°, X) peak mapping. The mapping area is ~2.8 × 3.0 cm^2^.

The Cd_0.9_Zn_0.1_Te_0.98_Se_0.02_ crystals were grown by the THM technique. The ingots are ~52 mm in diameter, weighing about 1.0–1.2 Kg (Fig. 1a). In general, the ingots are crack-free except near the Te-CZTS interface, as indicated by the arrows in Fig. 1a. The occurrence of these cracks is most likely due to the different thermal expansion coefficients of Te and CZTS leading to thermoelastic stress.

The composition vis. the band-gap of the CZTS ingot was found to be uniform for ~90% of the total length of the ingot (Fig. 1b,c), as measured by Energy Dispersive X-ray Analysis (EDAX). The wafer cut along the length of the ingot is shown in the inset of Fig. 1b. In view of the experimental accuracy of EDAX at ~±1 atomic %^22^, a high degree of uniformity in the composition was achieved for ~90% of the ingot length. Considerable depletion of Zn within ~1 cm near the Te-rich CZTS interface was observed (Fig. 1b). The band-gap of the material was estimated along the length of the ingot from the measured composition by EDAX, using the empirical formula by Brill *et al*.^23^, for the Cd_1−x_Zn_x_Te_1−y_Se_y_ quaternary compound at room temperature.

1 $${{\rm{Eg}}}_{(x,y)}=1.511-0.54{\rm{y}}+0.6{\rm{x}}\,(x,y\le 0.10)$$

where, 1.511 eV was considered to be the band gap of CdTe at room temperature. According to the Eq. 1, the theoretical band gap of Cd_0.9_Zn_0.1_Te_0.98_Se_0.02_ quaternary compound is about 0.011 eV lower as compared to Cd_0.9_Zn_0.1_Te.

Within the 1-cm region near the interface of the ingot, inevitable cracks occur for all THM grown CdTe-based ingots concomitant to the large thermal expansion coefficient difference between Te-rich CZT/CZTS alloy and CZT/CZTS. The high compositional uniformity along the length of the ingot ensures a higher yield as compared to CZT, where the zinc composition has a considerable wider variability. The compositional uniformity of THM-grown CZT with similar growth parameters was reported to be slightly more than one third of the total length of the ingot^24^. The incorporation of selenium is effective in modifying the segregation coefficient of Zn in CZTS ingot, so that it is closer to unity compared to CZT. To further investigate the radial compositional uniformity, we performed mapping of the low-temperature photoluminescence (PL) across the as-grown Cd_0.9_Zn_0.1_Te_0.98_Se_0.02_ wafer cut perpendicular to the ingot axis, cut about 2 cm below the interface. The spatial variation of the composition highly reflects the band-gap of the material. The PL peak energy positions are very sensitive to the band-gap, hence the composition of the material. The PL mapping was performed at Charles University, Prague. The typical PL spectrum (taken at 7 K) consists of a dominant acceptor-bound exciton (A°, X), a donor-bound exciton (D°, X) peak and the peak corresponding to the A-center (Supplementary Fig. 1). An area of about 2.8 × 3.0 cm2 was scanned for the two-inch-diameter as-grown Cd_0.9_Zn_0.1_Te_0.98_Se_0.02_ wafer cut perpendicular to the ingot axis. The spatial variation of the peak energy positions, viz. the map of peak energy at different positions of the scanned area with the step size of 1 mm, for the (A°, X) and (D°, X) peaks over the 2.8 × 3.0 cm2 area are plotted in Fig. 1d,e respectively. For both the (A°, X) and (D°, X) peaks, the spatial peak energy variations (∆E) are within 0.0015 eV over the scanned area of ~2.8 × 3.0 cm2. The observed small spatial variation of less than 0.0015 eV for both the (A°, X) and (D°, X) peaks confirms the excellent compositional uniformity for the as-grown quaternary compound Cd_0.9_Zn_0.1_Te_0.98_Se_0.02_.

**Figure 2:**
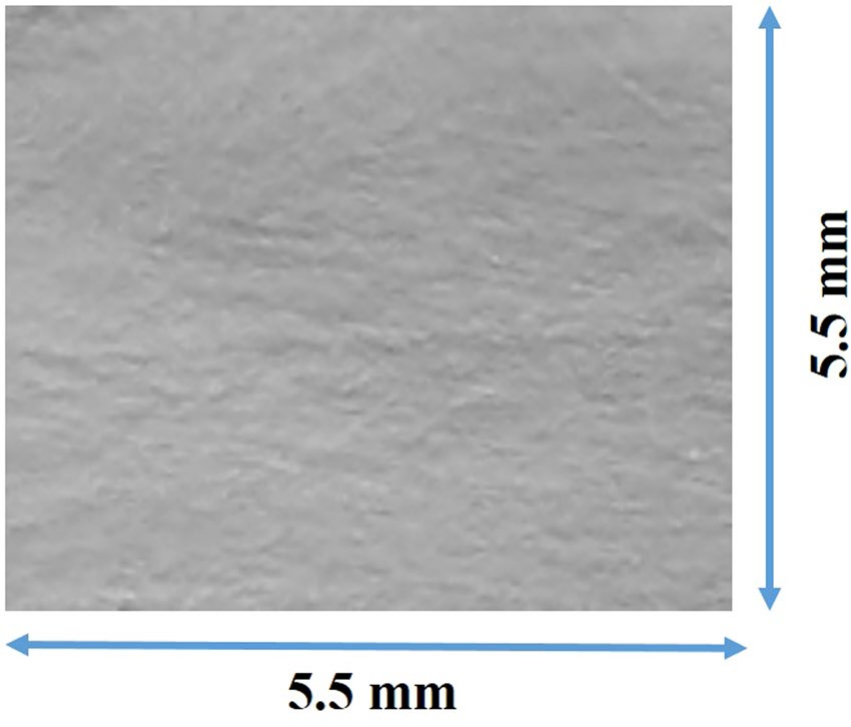
X-ray topographic image of an as-grown Cd_0.9_Zn_0.1_Te_0.98_Se_0.02_ sample. Exposed area: ~5.5 × 5.5 mm^2^.

An X-ray topographic technique was used to investigate the crystalline defects, such as sub-grain boundaries and their networks using the beamline 3.3.2 at Lawrence Berkeley National Laboratory’s (LBNL) Advanced Light Source (ALS). Figure 2 shows a representative X-ray topographic image of exposed area ~5.5 × 5.5 mm2 taken from the single crystalline sample of dimensions ~5.6 × 5.6 × 12 mm3, cut from a single grain from the as-grown Cd_0.9_Zn_0.1_Te_0.98_Se_0.02_ ingot. Any presence of sub-grain boundary and their network, are generally visible in X-ray topographic image by white and or black lines. The white lines correspond to the separation of the diffracted images of the adjacent sub-grains, while the dark lines correspond to the overlap of the diffracted images depending on the crystallographic tilt of the adjacent sub-grains. As illustrated, the sample is completely free from sub-grain boundary networks. For CZT, on the other hand are generally found to be highly decorated with sub-grain boundaries and their network^17^. For CdTeSe ingots grown by THM also we observed very few sub-grain boundaries and absence of sub-grain boundary network as revealed by X-ray topography^21^.

The occasional presence of an individual sub-grain boundary was observed in some regions of CZTS samples, but no sub-grain boundary network was detected. The absence of sub-grain boundary network and occasional presence of sub-grain boundaries in Se containing compounds such as CdTeSe and in the present case in Cd_0.9_Zn_0.1_Te_0.98_Se_0.02_, are very encouraging and elucidates that Se plays an important role in effective solid solution hardening of the matrix. Tanaka *et al*.^25^, reported decrease of lattice constant and also observed the effective solution hardening upon introduction of Se in CdZnTe matrix. The dislocation density was observed to be much lower and the absence of any cellular structure for Se containing compound^25^. The cellular structures are in general generate at the dislocation walls and are very common in CdTe and or CdZnTe compounds.

**Figure 3:**
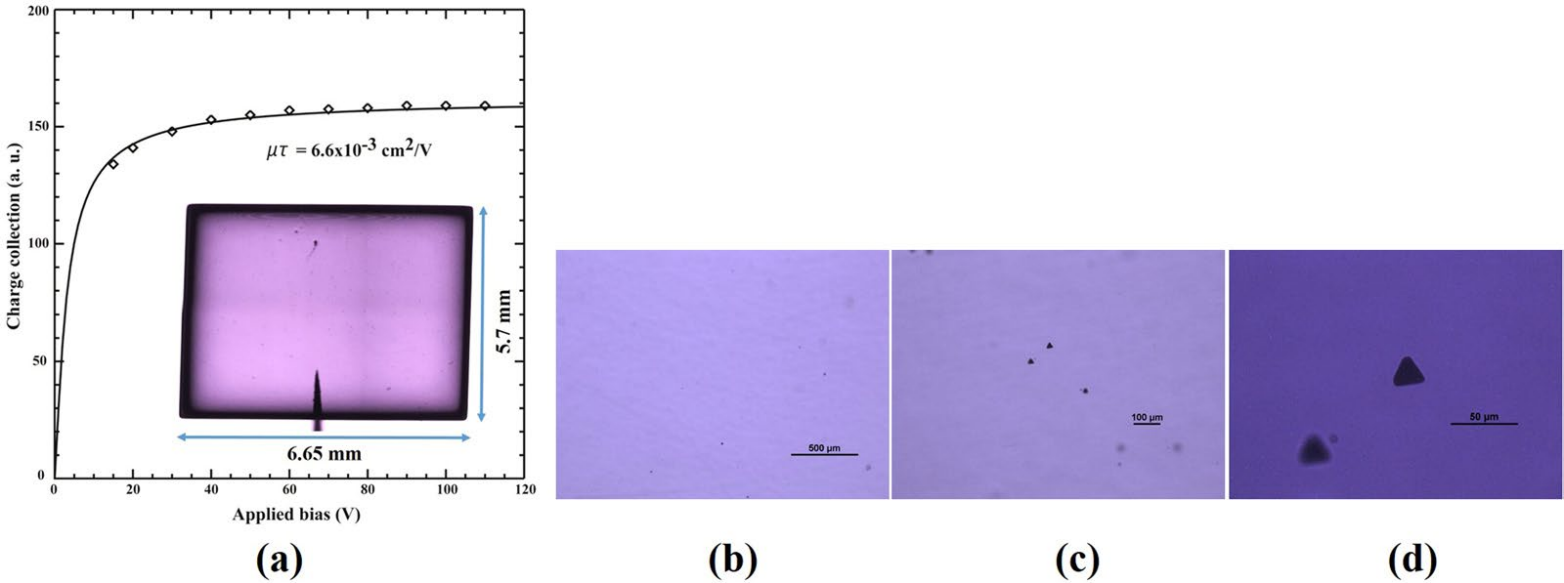
(**a**) Plot of the charge collection versus applied bias for the as-grown CZTS planar detector at room temperature. The 59.6-keV line was used from an ^241^Am source. The solid line indicates the Hecht fitting to extract the µτ value for electrons, the inset in Fig. 1a shows the IR transmission image of the whole sample of dimensions ~6.65 × 5.7 × 1.86 mm^3^, (**b**–**d)** high magnification IR transmission microscopic images of an as-grown CZTS sample showing triangular Te inclusions.

The charge-transport characteristics such as mobility-lifetime product of electrons (µτ)_e_ in the as-grown CZTS material was evaluated at room temperature for an Au/CZTS/Au planar detector. The (µτ)_e_ value was extracted by fitting the Hecht equation to the charge collection plot for an ^241^Am gamma source at an energy of 59.6 keV. The typical spectrum from an ^241^Am gamma source is shown in Supplementary Fig. 2, under the applied bias of −50V. The (µτ)_e_ obtained for the as-grown CZTS planar detector, ~6.65 × 5.7 × 1.86 mm3, was 6.6 × 10^−3^ cm2/V (Fig. 3a). The presence of Te inclusions was investigated by Infra-Red (IR) transmission microscopy. The IR transmission image of the entire sample is shown in the inset of Fig. 3a. As evident from the IR transmission image, very few Te inclusions are present in the as-grown CZTS. For comparison, very high concentrations of Te inclusions are generally reported to be present in the as-grown CZT grown by the same technique^26^. The high magnification IR transmission microscopic pictures are shown in Fig. 3b,c, depicting the presence of very few Te inclusions. The shape of the Te inclusions are triangular (Fig. 3c,d), which is typical for CZT-based material. The size of the inclusions was found to be about 16–20 µm with very low concentrations. The high magnification IR transmission microscopic images for different regions of the CZTS sample (Supplementary Fig. 3) reveal the absence of any large inclusions. A significant reduction of Te inclusions was confirmed through IR transmission microscopy as compared to as-grown CZT.

**Figure 4:**
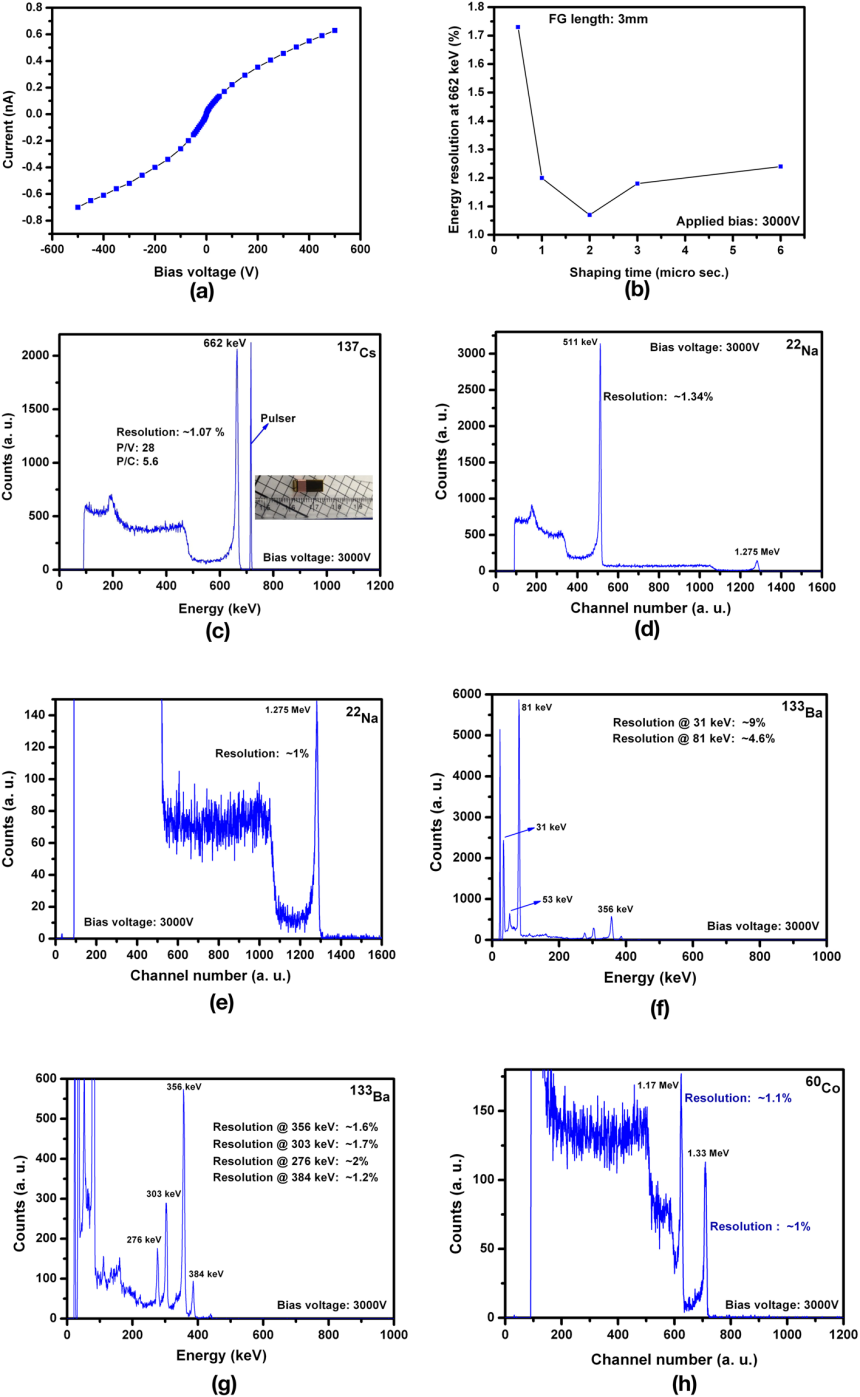
Current-voltage characteristics and gamma response for the Frisch grid detector fabricated from an as-grown CZTS ingot at room temperature. (**a)** Dark I–V characteristics of the detector sample at room temperature, (**b)** Detector response (energy resolution at 662 keV) at different shaping times, (**c)** Pulse height spectrum for the Frisch grid detector from a ^137^Cs source, the inset shows the photograph of the detector, (**d)** Pulse height spectrum for the Frisch grid detector for a ^22^Na source, (**e)** Zoomed version of the same spectrum to enhance the high energy peak, (**f)** Pulse height spectrum for the Frisch grid detector for a ^133^Ba source, (**g)** Zoomed version of the same spectrum to enhance the high energy peaks, and (**h**) Pulse height spectrum for the Frisch grid detector for a ^60^Co source showing very well resolved high energy gamma lines at energies of ~1.17 MeV and 1.33 MeV. The detector dimensions are: ~4.5 × 4.5 × 10.8 mm^3^.

The detector quality of the as-grown Cd_0.9_Zn_0.1_Te_0.98_Se_0.02_ material was appraised by fabricating detectors with a virtual Frisch grid geometry. The dark current-voltage relationship (I–V) at room temperature for an emblematic Frisch-grid CZTS detector with dimensions of ~4.5 × 4.5 × 10.8 mm3 shows very low leakage current (~0.63 nA) under an applied bias of 500 volts (Fig. 4a). The resistivity measured from the slope of the I–V plot for a ±1V (Supplementary Fig. 4) range was ~2.9 × 10^10^ ohm-cm, which fulfills the requirement for high resistivity detector-grade material. The fabricated detector with the Frisch-grid length of 3 mm was evaluated with various gamma sources with energies ranging from 31 keV to 1.33 MeV. The 662-keV gamma energy line from ^137^Cs source is generally considered as the standard in evaluating gamma detectors. The energy resolution at 662 keV of a detector with various shaping times was evaluated, and the best energy resolution was achieved with a shaping time of 2 µsec (Fig. 4b). Very high-resolution spectroscopic performance with energy resolution of ~1.07% at 662 keV was achieved (Fig. 4c) under an applied bias of 3000 V across the detector length (10.8 mm) with a shaping time of 2 µsec. The energy resolutions for the same detector at 662 keV with shaping times of 1 and 3 µsec obtained were within 1.2%. The peak-to-valley and peak-to-Compton ratios were 28 and 5.6 respectively (Fig. 4c), demonstrating excellent detector quality. The stability of the detector performance was examined by operating the detector at 3000 V and acquiring pulse height spectra at various time intervals ranging from 10 to 1000 sec. A very stable energy resolution (~1.1%), peak-to-valley and peak-to-Compton ratios for the measured 662-keV gamma line were achieved for the entire period of operation (Supplementary Fig. 5a,b). The sharp feature of the electronic pulse (Fig. 4c) indicates very low noise for the detector even under an applied bias of 3000 V. The 511-keV and 1.275-MeV gamma rays from a ^22^Na source were measured by the detector showing an excellent energy resolution of 1.34% and ~1% respectively (Fig. 4d,e). All the gamma lines from a ^133^Ba source including the 31-keV X-ray line were successfully identified by the as-grown CZTS detector with very high energy resolution (Fig. 4f,g). The energy resolution at 31 keV was ~9%, while for the 81-keV and 356-keV peaks, the corresponding values are 4.6% and 1.6% respectively. The CZTS detector resolved high energy gamma lines at energies of 1.17 MeV and 1.33 MeV from a ^60^Co source with energy resolutions of ~1.1% and 1.0% respectively (Fig. 4h). The detector quality of the as-grown CZTS material is comparable to very high quality commercially available CZT detectors. All the pulse height spectra from different sources presented here are as-measured, i.e., without any charge-loss corrections.

## Conclusions

The efficacy of adding Se to the CZT matrix for radiation detector applications has been successfully demonstrated. Selenium was effective in modifying the segregation coefficient of Zn, resulting in higher compositional homogeneity along the length of ingots as opposed to CdZnTe. The higher compositional homogeneity offers remarkable potential to increase the overall production yield of CZTS material at a lower cost. Selenium was seen to be very effective in reducing the secondary phases (Te inclusions) in the grown CZTS ingots and in lattice hardening resulting in CZTS ingots free from sub-grain boundary networks. The secondary phases and the sub-grain boundary networks are the major issues affecting the performance and cost of CZT detector technology. Both types of defects are deleterious and are known to hinder the charge transport characteristics, resulting in severe degradation of the detector performance. The addition of Se was found to be very effective in resolving the decade-long issues suffered by CZT. The reduced density of Te inclusions and the absence of sub-grain boundary networks in CZTS material makes it a promising approach to increase the yield of high-quality detectors. Well-resolved high-resolution gamma lines in the energy range of 31 keV to 1.33 MeV were observed for virtual Frisch grid as-grown CZTS detectors, demonstrating the excellent material properties, including high compositional homogeneity, a very low concentration of Te inclusions and the absence of sub-grain boundary networks.

## Methods

The Cd_0.9_Zn_0.1_Te_0.98_Se_0.02_ compound was first synthesized from a stoichiometric amount of 6N purity Cd_0.9_Zn_0.1_Te from 5N Plus Inc. and 6N purity CdSe from Azelis Inc. The THM growth was carried out from 6N purity Te-rich solution with In as the dopant. Both 6N purity Te and In were procured from Alfa Aesar. Prior to synthesis and growth by THM, the inner walls of the ampoules were coated with carbon by cracking spectroscopic grade acetone at ~900 °C, followed by annealing the coated ampoules at ~1150 °C for one hour. The ampoules were then loaded with the required amount of materials and sealed under dynamic vacuum of ~2 × 10^−6^ torr. All the CZTS crystals were grown with 52-mm inner diameter using conically tipped, high purity quartz ampoules. The THM growth runs were carried out in a three-zone furnace with growth parameters similar to those used for CZT growth^27^. The ingots were grown with a lowering rate of 3–3.5 mm/day, and the temperature gradient near the interface was maintained between 10–15 °C/cm. After completion of the growth, the ingots were cooled down to room temperature at a rate of ~100 °C/day.

The grown ingots were cut into pieces using a diamond-impregnated wire saw to different sizes for various characterizations. The cut samples were then polished with successive grit size SiC paper and finally polished with 0.05-µm alumina suspension on felt pad to achieve mirror-finish surfaces. IR transmission microscopic studies were carried out using a Nikon, Eclipse LV 100 Microscope. Compositional analyses were performed using a Jeol 7600 electron microscope equipped with Energy Dispersive Spectroscopy. White Beam X-ray Diffraction Topography (WBXDT) measurements were carried out at LBNL’s ALS Beamline 3.3.2 with an X-ray beam energy ranging from 4 keV to 25 keV. The X-ray topographic measurements were carried out in reflection mode. The schematic of the experimental setup has been illustrated in our previous publication^21^. The samples used for the topographic experiments were cut from single crystalline grains. The low-temperature PL mapping was carried out at Charles University using a Radius (Coherent) diode laser for excitation of the sample, with the excitation energy of 1.95 eV having power of 25 mW. The beam area used for excitation was ~0.25 mm2. The sample was raster scanned with the step size of 1 mm. For both topographic and PL mapping experiments, the samples were etched in 2% bromine methanol solution for two minutes to remove any possible damaged layer that might be introduced during the polishing process. A Keithley 6487 picoammeter/voltage source was used for the dark current-voltage measurements.

## Supplementary information

Evaluation of CdZnTeSe as a high-quality gamma-ray spectroscopic material with better compositional homogeneity and reduced defects

## Data Availability

The data supporting this study are available from the authors on reasonable request.
