# Supplementary material for: Evaluation of CdZnTeSe as a high-quality gamma-ray spectroscopic material with better compositional homogeneity and reduced defects
Source: Sci Rep. 2019 May 13;9:7303. doi: 10.1038/s41598-019-43778-3 (PMC6513868; doi:10.1038/s41598-019-43778-3)
Supplement: Supplementary file 1 — Evaluation of CdZnTeSe as a high-quality gamma-ray spectroscopic material with better compositional homogeneity and reduced defects [file 41598_2019_43778_MOESM1_ESM.pdf]

Supplementary Information

Evaluation of CdZnTeSe as a high-quality gamma-ray spectroscopic material with better compositional homogeneity and reduced defects

Utpal N. Roy[1], Giuseppe S. Camarda[1], Yonggang Cui[1], Rubi Gul[1], Ge Yang[1*], Jakub Zazvorka[2], Vaclav Dedic[2], Jan Franc[2] and Ralph B. James[1**]

[1]Brookhaven National Laboratory, Upton, NY 11973, USA
[2]Institute of Physics, Charles University, Ke Karlovu 5, Prague 121 16, Czech Republic

[*]Present address: North Carolina State University, Raleigh, NC, 27695-7909, USA
[**]Present address: Savannah River National Laboratory, Aiken, SC 29808, USA

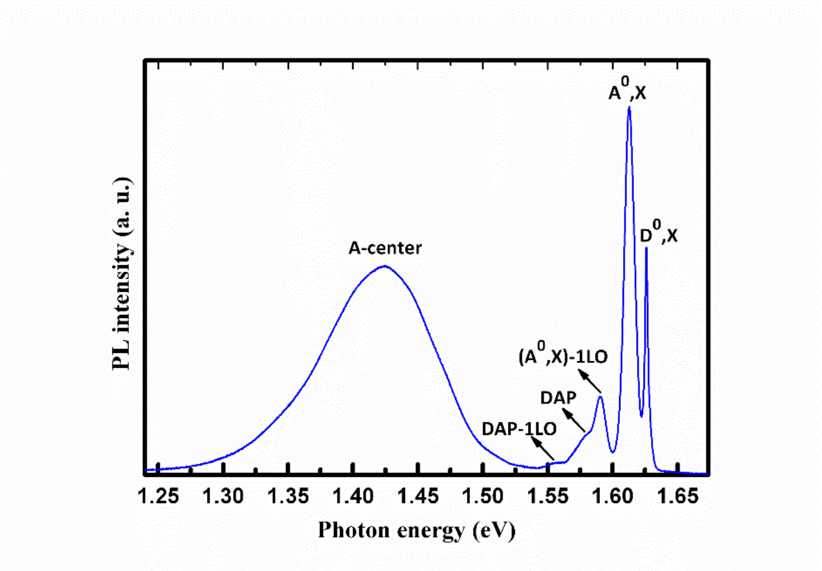

Supplementary Fig. 1. Low temperature (7 K) photoluminescence spectrum of $Cd_{0.9}Zn_{0.1}Te_{0.98}Se_{0.02}$ grown by THM.

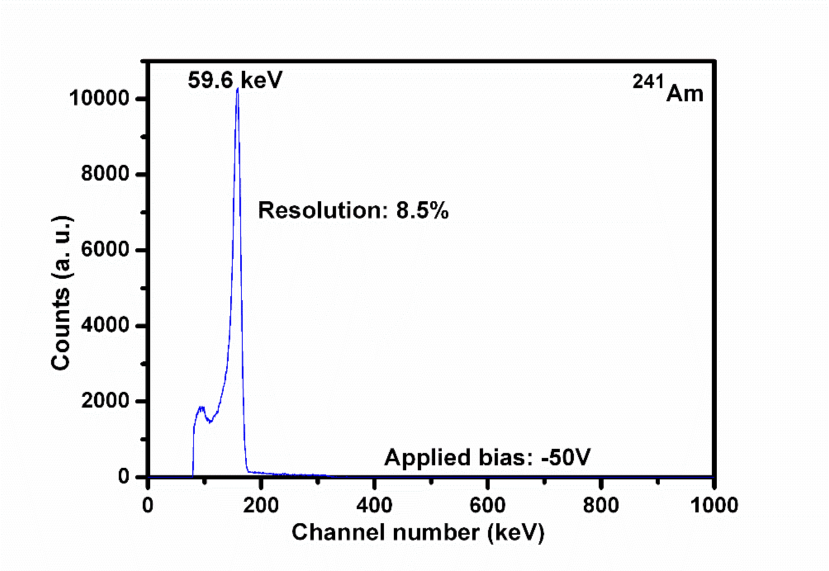

Supplementary Fig. 2. Pulse height spectrum for the planar detector from an ^{241}Am source. The detector dimensions: 6.65x5.7x1.86 mm^3.

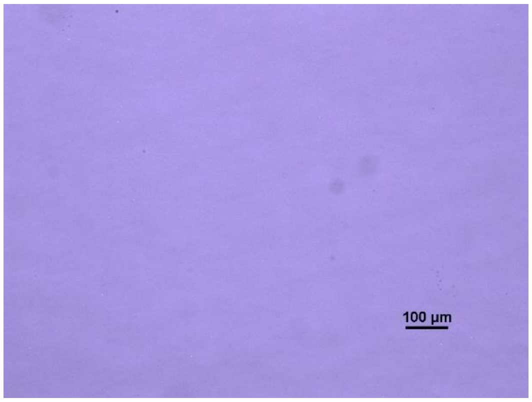

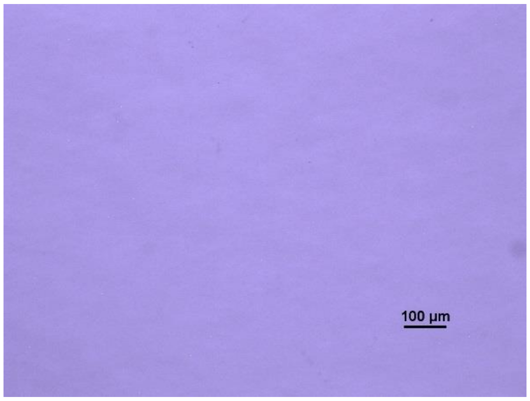

Supplementary Fig. 3. High magnification IR transmission microscopic images of an as-grown CZTS sample.

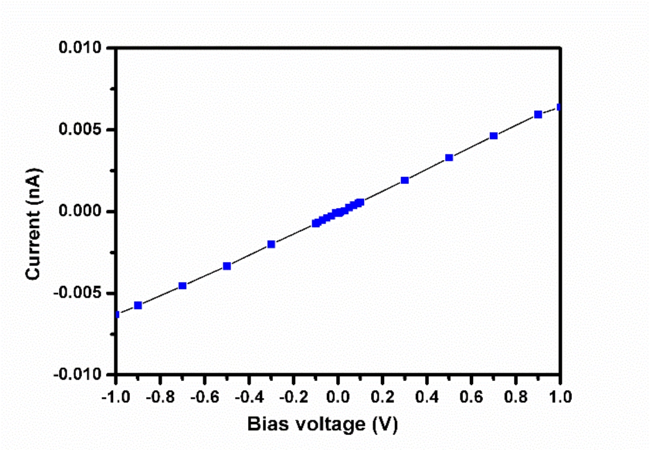

Supplementary Fig. 4. Dark current-voltage characteristics at room temperature for a ±1V range.

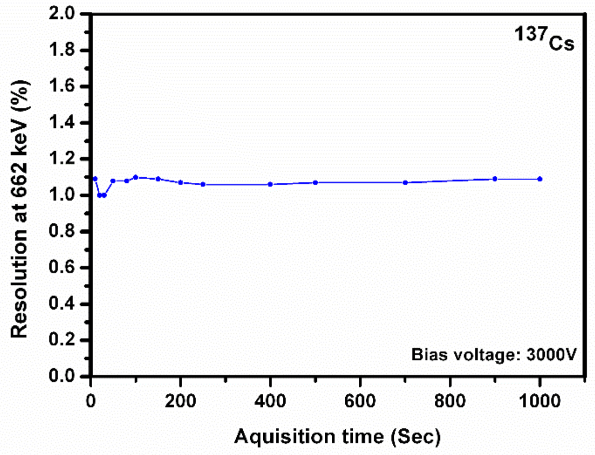

(a)

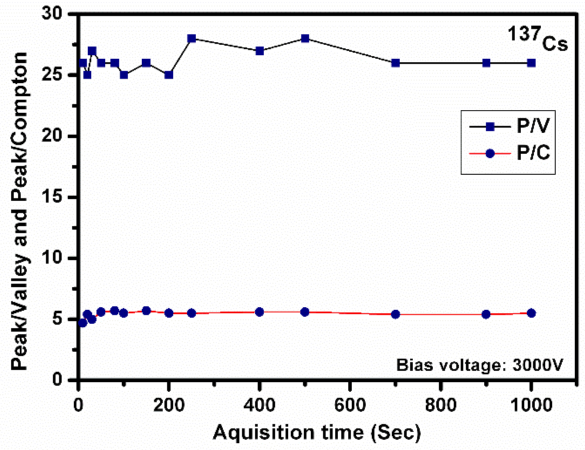

(b)

Supplementary Fig. 5. a) Energy resolution at 662 keV with acquisition time, and b) P/V and P/C of the 662 keV with acquisition time for the as-grown Frisch grid detector. Detector dimensions: 4.5x4.5x10.8 mm^3.
